# Diagnosis of Diseases of the Urinary System

**Published:** 1909-07-17

**Authors:** J. G. Pardoe

**Affiliations:** Senior Assistant-Surgeon St. Peter's Hospital; Assistant-Surgeon West London Hospital


### Hospital Clinics.

### DIAGNOSIS OF DISEASES OF THE URINARY SYSTEM.

By J. G. PARDOE, M.B., C.M., F.R.C.S.; Senior Assistant-Surgeon St. Peter's Hospital; Assistant-Surgeon West London Hospital.

(A Lecture delivered at the Post-Graduates' College, West London Hospital, on June 28, 1909.)

This afternoon I propose to talk to you about some methods of diagnosis of diseases of the urinary system. Some methods, of course, are perfectly simple and applicable by everybody, but there are others which call for the aid of the skilled diagnostician. Let us begin with the consideration of the diagnosis of certain diseases of the urethra. The most common of all the diseases affecting the urethra is chronic urethritis, and this is the disease which is treated in a more haphazard way than most others; as a rule simply for want of proper diagnosis. The urethra, for clinical purposes, can be divided into a deep and a superficial part, or, in other words, an anterior and a posterior part. Any infection behind the compressor urethræ muscle cannot be cured by the use of ordinary anterior injections with a small syringe. Many of these cases of chronic urethritis are due to posterior infection—infection of the mucous membrane of the deeper urethra, or of the prostate itself. It must be our first object to find out whether the infection is limited to the anterior urethra or has also involved the posterior.

The simplest method, applicable by anybody without special apparatus, is that known as the four-glass test. With an ordinary irrigator can, to which is attached four or five feet of tubing and a proper nozzle, the anterior urethra is thoroughly washed out with sterile water or with boracic solution. In order thoroughly to wash out the anterior urethra sufficient force must be used, but one must be careful not to use too much force. If too much force is employed it will be found that fluid has been sent back into the bladder, thereby vitiating the test. I usually have the patient standing for this test, his trousers down to his knees, and a bowl held beneath the penis. The nozzle is a conical one, so that if pressed into the meatus the meatus is blocked. By means of alternate pressure and release the anterior urethra first balloons and then relaxes. In that way it is wholly flushed out, and the washings are placed in a glass. The patient then passes a little urine which is put into a second glass, and passes a little more which is put into a third glass. We then put the patient in a kneeling position on the couch, and with our forefinger in the rectum massage out the contents of the prostate and the seminal vesicles. After this the patient stands up and passes the urine into the fourth glass. We thus have the contents of four glasses. In the first place, the anterior urethra as far back as the compressor urethræ is washed, therefore the contents of the first glass will be mainly débris from the anterior urethra. In the second glass containing the first urine we shall have anything which comes from the superficial mucous membrane of the deep urethra. The third glass contains the bladder contents uncontaminated from the urethra; and the fourth glass the bladder contents, together with the contents of the follicles of the prostate and the seminal vesicles. In this way we are furnished with a direct means of ascertaining the extent and depth of the infection.

I am not speaking about treatment this afternoon, but simply about diagnosis. I may say, however, that if there is definite infection of the prostatic follicles, ordinary injections in the penile and bulbous urethra will be perfectly futile. The history of such a case is very common. The patient has had what he calls gleet for a considerable number of weeks or months. After a certain amount of treatment it disappears, but on seminal emission or sexual intercourse the discharge returns. The infection has been carried from the depths of the prostate into the urethra and has again caused catarrhal urethritis. In making an examination it is necessary always to have the patient with a very long retention of urine. The best plan is to prohibit him from passing water when he rises from bed in the morning, so that we have the whole night's urine to go upon. Moreover, he should not have had a motion before coming for examination, otherwise a certain amount of the prostatic contents may be squeezed into the urethra. It is also very important that he should not have had a seminal emission during the night. These little precautions should be observed, or the test will not be satisfactory.

So much, then, for this method of diagnosis in the case of chronic urethritis. It is almost unnecessary to speak about the urethroscope. Chronic urethritis, however, is not always dependent upon an infection of the deep part of the urethra. Many cases are entirely confined to the anterior urethra. Very frequently the cause is infection of the glands of Littré. These may not be felt on taking the urethra between finger and thumb, but the urethroscope will show the glands at once. The glands mature in just the same way as an acne follicle in the skin will mature, forming a kind of boil. One of these small glands comes to a point, ruptures, and the urethra is again infected with gonococci or other micro-organisms. The urethroscope is practically the only means of detecting this condition. In the many cases which have been cured when steel bougies have been used the cure has been due frequently to the rupture of these glands by the large metal instruments, thereby setting free the gland contents and allowing them to heal.

I want now to pass on a little further and consider the diagnosis of some conditions in the bladder. Supposing that we take the cardinal symptoms of urinary disease—pain, connected or unconnected with micturition; frequency of micturition; hæmaturia; and pyuria—all these symptoms, alone or in conjunction, may give rise to a misleading diagnosis. Let me give you an instance. Quite recently I have seen a case of urinary disease which has mimicked a variety of other conditions and has led to a series of grave surgical mistakes. Twenty-five years ago the patient was delivered of a child under very difficult circumstances and without adequate assistance. She never felt well after the confinement, and particularly felt pain after passing urine. A year or two after her confinement she was operated upon for these pains, and her left ovary was removed. I have no information as to the state of the ovary. The pain still continued, however, and subsequently the right ovary was removed. Here, again, the condition of the ovary is not stated. Seven or eight years ago, still complaining of pains in connection with the bladder and pelvic organs, she underwent an exhaustive examination; another laparotomy was done, and this time the uterus, which was said to be pressing on the bladder, was removed. I have no information further than this, but of course we know that the healthy uterus does not press upon the bladder and cause these symptoms. There was no history of fibroids in this connection. Well, the pains still continued, and the woman resigned herself to endure them to the end of her days, but a year or so ago she felt that they were too much to endure, and again sought advice. They got rather nearer the mark this time, for they said that she had chronic Bright's disease, and must not have anything more done. She had intermittent frequency of micturition; for a week or two the frequency would be very great, and for a further period less so. There was always pain at the close of micturition; never any hæmaturia; slight backache. The cystoscope showed a considerable efflux of pus from the left ureter, and on calling to our assistance the *x*-rays it became evident at once that she had a large stone in the kidney of her left pelvis. This stone I removed the other day, and since then she has had no pain in the pelvis or in connection with her urinary system. One must not be dogmatic in such a case, particularly in the absence of more detailed information, but with this large, slow-growing oxalate stone in the kidney it does look as if, had it been possible to employ cystoscopy and radiography at the beginning, that stone would have been removed, and the patient spared many years of ill-health and pain.

The point of the above case is that renal conditions may simulate bladder diseases, and that bladder disease may simulate renal conditions. And it leads me to the axiom, or the postulate, whichever you will, that no kidney should be operated on until a careful cystoscopy has been made. That is a counsel of perfection, but I always carry it out in my own practice. The instance I have just given was one of a renal stone simulating bladder trouble. Before passing on to consider the bladder I will describe one more case. A young woman, about thirty years of age, came into the hospital with profuse hæmaturia. So profuse was it that she appeared to be almost dying. The bladder filled rapidly with clots. The bleeding was temporarily stopped by ordinary means, and on examination an immense tumour was found in the left loin. The patient had complained of severe pain in that region for three or four months. The natural inference was that there was a rapidly growing malignant growth of the kidney. A cystoscopy was done and showed a villous growth hanging to a long pedicle attached to the left ureteric orifice. The operation was a dramatic one, for this large tumour acted like a pricked bubble; there was an immense gush of urine which overflowed on to the table and the floor when the villous growth was removed, and the kidney tumour immediately went down. Without the cystoscope that kidney would have been operated on and probably removed, and nothing whatever would have been done to the villous tumour.

There are certain conditions in which the use of a cystoscope is essential. One of these lies in making a right choice for the treatment of stone in the bladder. Everyone is agreed at the present time that the operation of choice for treatment of stone in the bladder is litholapaxy—*i.e.* crushing the stone and removing the fragments at one sitting. Litholapaxy is an easy operation under favourable conditions, when the bladder is not contracted and the stone not too large, and when there is no interference from stricture of the urethra or great enlargement of the prostate. But where any difficulties are present the young litholapaxist may be better advised to do the ordinary supra-pubic section and remove the stone through an incision. Litholapaxy, moreover, in some cases presents serious dangers if a sacculus is present, and the stones are in the sacculus, or, it may be, in the bladder, covering up the sacculus to some extent. Sacculi have an unpleasant habit of opening at about the base of the bladder. Let us suppose that a sacculus has its opening between the two ureters. A fairly big stone may form in certain bladders, resting at the base, and may be caught in the lithotrite and found to be perfectly movable in the bladder. That stone is crushed and the sharp and jagged fragments drop into the sacculus, and the evacuator fails to suck them out. Ulceration is set up and the course of events is sometimes very tragic.

There is a difference between an acquired sacculus and a congenital one. The acquired sacculus is usually due to straining or back pressure in making urine. In this way a sacculus is gradually formed, the wall of which is merely mucous membrane with a little fibrous tissue. A congenital sacculus, on the other hand, may be a kind of accessory bladder. Into an acquired sacculus with its thin mucous membrane wall fall fragments of stone. A lot of urine leaks out, and a serious condition is set up. The recurrence of grave symptoms after an apparently successful operation is a great disappointment to the surgeon. After the straightforward litholapaxy the patient is put back to bed and for a day or two is quite comfortable. Then he begins to complain of deep pelvic pain, and within a short time there is rigidity above the pubes, generally on the right side, pain down the leg following the course of the anterior-crural nerve, a dry tongue, and perhaps a rigor. I have seen this accident occur eight times altogether, so that it cannot be called an uncommon one. Two of the cases were my own, and both recovered, but only after a most serious illness, enormous incisions, free drainage, and a very long convalescence. Of the other six cases four recovered and two died. Therefore this is a serious complication and a distressing and calamitous one. I am thus led to lay down the rule that no stone should be crushed in the bladder until after a careful cystoscopy, wherever that is possible.

Perhaps the most important revolution in the diagnosis of urinary conditions has been in surgical conditions of the kidneys. This has been brought about by the use of two instruments—the intravesical segregator and the catheterising cystoscope. The most striking results have been gained in connection with tuberculosis of the kidneys. The statistics given by Israel, of Berlin, in the latest edition of his book show the immense progress which has been made in renal surgery since the introduction of these careful, systematic methods of diagnosis. In the first place we have to ascertain in many cases which kidney is diseased, if, indeed, both are not affected, and in the second place we have to make sure whether the kidney which is apparently not grossly diseased is of sufficient functional activity to carry on the needs of the body when the other has been removed. If in any case diagnosis of an accurate nature is required, it is in tuberculosis of the kidneys. The introduction of an ordinary examining cystoscope into the bladder will enable the surgeon to gain sufficient experience to appreciate the character of the efflux from either kidney with very little practice. When severe disease is present the pus is sometimes so thick that when squeezed from the ureteric orifice it is like lanolin from a tube. At times it is not so thick, and in certain cases the efflux stops during cystoscopy—a most annoying thing. Apparently the very act of passing instruments into the bladder inhibits the stream from the kidneys; therefore we either segregate by the segregator or by the passage of the ureteric catheter. I much prefer the latter method.

In addition to these we have other methods at our command which tell us of the functional activity or competence of the two kidneys—namely chromatoscopy and the production of artificial diabetes. In the first method one uses either methylene blue or indigo carmine. By giving the patient two or three grains of methylene blue in pills at intervals of several hours before the operation, or by injecting it hypodermically half an hour before the examination, the urine from normal kidneys is deeply and equally tinged with the pigment. But in the case of an incompetent kidney the colour is either exceedingly pale or is entirely absent. This is a most valuable method, because sometimes a kidney which is discharging quite a clear stream of urine to the eye is anything but competent for its work, particularly in tuberculosis. I remember a somewhat tragic case which instanced the truth of that most plainly. A youngish woman had tuberculosis of the bladder, and obvious discharge of pus from one kidney. Her clinical history was that she had first of all suffered from pain in one lumbar region, corresponding to this pus-discharging kidney, and then her symptoms of bladder trouble developed. The kidney was removed by nephrectomy. Well, she never passed any more water, or, at least, she passed very little, and lived the usual ten or twelve days with complete suppression. At the post-mortem examination her other kidney was found to be grossly infected with tubercle which had not broken down. The whole pelvis was studded with minute tubercle. My experience of the methylene blue test in such a case as that leads me to believe that if it had been done the side where the nephrectomy was carried out would have shown no trace of the pigment, and the other side a very slight trace.

The other method is more difficult—the estimation of sugar artificially produced by the hypodermic injection of phloridzin. The matter is too long to be entered into in detail, but the fact remains that the sugar recovered from healthy kidneys is practically constant on the two sides, whereas even a small interference with the functional activity of the kidney greatly influences the output of sugar.

Another important point in connection with this diagnosis of urinary disease is the question of bacterial infections of the urinary system. Too frequently one hears the diagnosis made of cystitis—cystitis pure and simple. Having said that, the presumption is that all has been said that needs to be said. Nowadays we are not content with that, and we have to call bacteriology to our aid. Of late years a very great variety of bacterial invasions of the bladder have been recognised and adequately treated. Instead of saying, "Yes, that is a cystitis; let us wash it out for local treatment and give urinary antiseptics for general treatment," we now find out the particular micro-organism concerned and treat the case accurately. A case in point is the infection with bacillus coli, and a very great distinction must be drawn between the acute coli cystitis and the chronic one. Acute infection with bacillus coli very often follows an attack of influenza, and a pure culture of coli is found to grow from the urine. In such a case local interference is very inadvisable. The catheter should not be passed, but the patient should be put to bed and given hot baths and urinary sedatives, and as a rule he will come right. Vaccine is of no benefit in such cases. But in other patients, who come with a story of years of suffering and infection of urine, the case is different. Though they may have tried every drug from A to Z, yet the urine is still cloudy, and one commonly finds a mixed infection of bacillus coli accompanied by staphylococcus or streptococcus. In such cases one can sometimes remove the infection of the secondary organism, whereas the coliform infection persists, and then vaccine sometimes does enormous good.

Tuberculosis of the bladder is one of the most insidious diseases we know. It commences so very quietly, so little alarm is caused. Possibly some undue frequency of micturition is the earliest symptom. The patient does not notice it during the day, but he does notice it at night; he may have to get up once or twice in the night for that purpose, even in warm weather. In such a case as that the urine may be very nearly or quite clear; there may be no blood or pus to the naked eye, but if the urine is carefully examined bacteriologically it will very often reveal the presence of acid-fast bacilli. It is not uncommon for the first hint of the condition to be given by the examination for life insurance. I had such a case the other day. It was that of a doctor who went to be examined for life insurance. It was rather a strict examination, and they noticed—what he had never previously noticed—that his urine was a little muddy-looking. He asked a colleague to examine it, and definite tubercle bacilli were found. There were no symptoms of any kind whatever. He went to a sanatorium, had treatment with tuberculin, put on nearly two and a half stones in weight, and then returned to work. Almost immediately he had a fresh invasion of tubercle, and a nephrectomy was done. He had been passing plenty of tubercle bacilli in the urine and knew nothing whatever about it. Therefore, I insist that when cystitis is obviously going on a bacteriological examination should be made. It will at least give timely warning of the necessity for surgical or other treatment.

